# Current Status and Future Prospects on Nanodelivery Systems Targeting the Small Intestine for Absorption of Bioactive Substances

**DOI:** 10.3390/foods14183234

**Published:** 2025-09-17

**Authors:** Hong Zhang, Mengjie Su, Yu Zhang, Qiuxia Feng, Yuntao Liu, Zhen Zeng, Qing Zhang, Zhengfeng Fang, Shanshan Li, Hong Chen

**Affiliations:** College of Food Science, Sichuan Agricultural University, No. 46, Xinkang Road, Yucheng District, Ya’an 625014, China

**Keywords:** targeted nanodelivery systems, small intestine, bioactive substances

## Abstract

The undesirable properties of bioactive substances (such as poor solubility and low stability) and various barriers in the gastrointestinal tract (gastric acid, digestive enzymes, mucus and intestinal epithelial cells) hinder their absorption and utilisation by the human body. Nanodelivery systems have been proven to effectively address the above problems, particularly targeted nanodelivery systems, which have more advantages in improving the bioavailability of bioactive substances. However, many studies have not included all barriers. Furthermore, given that the small intestine is the main site for the absorption of bioactive substances in the human body, this review primarily discusses targeted nanodelivery systems designed for the gastrointestinal barrier and summarises how to construct a nanodelivery system that can resist the adverse effects of the gastrointestinal tract and target the small intestine for the absorption of bioactive substances. This paper proposes that the ideal system is the active targeted nanodelivery system that targets enterocytes and its future development trend is discussed. This review aims to provide new insights for the rational design of nanodelivery platforms that efficiently target the small intestine and promote the absorption of bioactive substances, as well as promote the development of fields such as personalised nutrition and nutritional intervention.

## 1. Introduction

The harsh environment of the gastrointestinal tract makes it difficult for most bioactive substances to be absorbed and utilised by the human body through direct ingestion [1,2,3]. Increasing the bioaccessibility and bioavailability of bioactive substances has been one of the topics of concern in the field of delivery systems. Bioaccessibility refers to the extent to which an bioactive substance can be absorbed and utilised in the gastrointestinal tract as it is released from the matrix; as bioaccessibility increases, so does bioavailability (usually referring to the proportion of a bioactive substance that enters the bloodstream) [4,5]. Numerous studies have shown that nanodelivery systems have the advantage of masking undesirable flavours, enhancing solubility and physicochemical stability in vivo and in vitro, controlling release and improving delivery efficiency of bioactive substances; these benefits can help overcome inherent deficiencies within bioactive substances themselves and the harsh environment of the gastrointestinal tract [6,7,8,9]. Recently, technological advances in food and pharmaceutical fields have led researchers to design various novel nanodelivery systems for bioactive substances, including nanoliposomes, nanomicelles, polymeric nanoparticles, nanogels and nanoemulsions. Each type of nanodelivery system differs in the loading modes (e.g., interaction forces) and effects (e.g., encapsulation efficiency, loading efficiency and stability) of bioactive substances. Their respective advantages and disadvantages can also be learnt from some recently published literature [10,11,12,13]. Compared with non-targeted nanodelivery systems, targeted nanodelivery systems are more advantageous in enhancing the enrichment of bioactive substances at the target site and improving their bioavailability. Oral administration is the predominant route of bioactive substances delivery due to its inherent advantages in terms of safety, convenience, high patient compliance, and cost-effectiveness compared to other routes [6]. The small intestine is the main site for the absorption of bioactive substances in the human body. In contrast, the small intestine offers a more favourable environment for bioactive substances absorption. Its pH is less acidic than the stomach and enzymes better suited for bioactive substances digestion and absorption [14]. In addition, compared with the other compartments of the GI tract, the peristalsis of the small intestine is also more regular, which makes oral bioactive substances delivery more predictable and consistent [15]. Therefore, this review mainly focuses on the research progress on passive and active oral nanodelivery systems targeting the absorption in the small intestine. By reviewing the absorption barriers in the gastrointestinal tract and the corresponding delivery strategies, we expect to provide ideas for the design and construction of nanodelivery systems for efficient absorption via the small intestine.

We conducted a literature search spanning the last decade using the Web of Science and ScienceDirect databases, primarily employing combinations of keywords such as Nano, Delivery, Oral, Target, and Intestine. Given the diversity of nanodelivery systems covered in this review, additional keywords were incorporated as needed, with priority given to the references with an impact factor greater than 5. For example, “pH-sensitive” was included for pH-sensitive nanodelivery systems. In addition, it should be mentioned that we particularly prioritised studies focused on delivering food-derived bioactive substances. Although the studies listed in this paper include nanodelivery systems for drug models such as insulin, their design concepts can provide valuable references for delivering food-derived macromolecular bioactive peptides or proteins and other small molecules.

## 2. Adsorption Barriers

**Figure 1:**
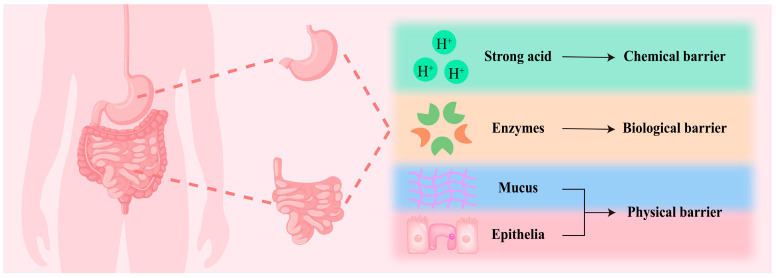
Gastrointestinal barriers that bioactive substances need to pass through before being absorbed by the small intestine.

Before being absorbed in the small intestine, a bioactive substance must pass through the oesophagus, stomach and duodenum of the upper gastrointestinal tract, as well as the jejunum and ileum of the lower gastrointestinal tract. As illustrated in Figure 1, their chemical (gastric acid), biological (enzymes in the gastrointestinal tract) and physical (mucus and small intestinal epithelial cell layers) barriers combine to prevent the bioactive substance from being utilised by the human body, which ultimately leads to reducing bioavailability [16,17]. Among them, chemical and biological barriers primarily impact the stability of bioactive substances, whereas physical barriers decrease their bioavailability by preventing penetration and absorption.

### 2.1. pH and Enzymes in the Stomach and Small Intestine

The stomach has a low pH level (1.5–3.5) and an abundance of digestive enzymes; although it protects the human body from microorganisms/xenobiotics, its specific pH environment alone could expose bioactive molecules to degradation/decomposition [18]. The small intestine is generally considered the most favourable region of the gastrointestinal tract for bioactive substance delivery because of its long transit time, extensive surface area, high epithelial permeability and subsequent availability for systemic absorption [19,20]. Its pH gradually increases from about 6 in the duodenum to approximately 7.4 in the terminal ileum [21]. Furthermore, the small intestine contains pancreatic enzymes including lipase, trypsin, amylase and peptidase. These enzymes are synthesised by the pancreas and secreted into the intestinal lumen, and they are particularly abundant in the upper part of the small intestine (i.e., the duodenum) [22]. These enzymes in the small intestine, together with the enzymes in the stomach, form the biological barrier before absorption in the small intestine.

### 2.2. Mucus

Mucus is a viscous, semi-permeable matrix secreted by epithelial cells that permits the exchange of nutrients, water, gases and hormones while restricting entry of most bacteria and pathogens because of its steric hindrance and adhesive properties [23,24]. It is reported that average pore size within small intestinal mucus gels is approximately 211 nm, with 90% ranging between 100 and 300 nm [25]. The primary components of the mucus layer include water (up to 95% by weight), mucins (typically not exceeding 5%), inorganic salts (about 1%), carbohydrates and lipids; mucins (accounting for over 80% organic components of the mucus) control the gelatinous structure of the mucus [26]. Mucins are a large and complex family of glycosylated proteins secreted by goblet cells, and the O-glycosylation process present in their ‘mucin structural domain’ not only confers mucins with the ability to bind and dissolve in water for gels formation, but also generates a glycan shell that can hide the protein core of mucins and protect them from degradation by endogenous proteases [27]. The protein backbone of mucins, which is rich in thiol groups and contains hydrophobic and charged structural domains, also includes sulphate groups that together with the carboxyl groups in sialic acid, confer a net negative charge to mucins [28]. Overcoming the mucus barrier aims to increase close contact of bioactive substances with the absorptive membrane and improve their absorption by enhancing the interaction with intestinal epithelial cells [29].

### 2.3. Intestinal Epithelial Cells

Bioactive substances also need to overcome the intestinal epithelial cell layer before entering systemic circulation. This layer consists mainly of tight junctions between cells and a monolayer of columnar epithelial cells with different characteristics and receptors, including enterocytes (the main absorptive cells, accounting for 80–90% of epithelial cells), goblet cells (which produce mucins to form the mucus layer covering the epithelial surface), microfold cells (also known as M cells, which are specialised epithelial cells situated in Peyer’s patches and are part of the intestine-associated lymphoid tissues) and Paneth cells (which secrete specific antimicrobial proteins that selectively bind to and kill bacteria) [16,30,31,32].

**Figure 2:**
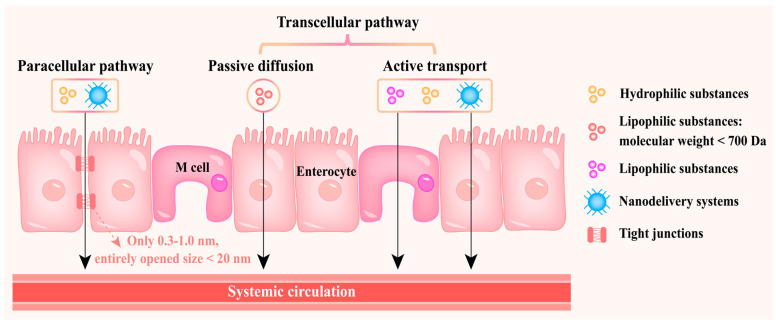
The way in which nanodelivery systems and substances with different properties pass through the intestinal epithelial barrier.

Nanodelivery systems and substances with different properties have various modes of transport in intestinal epithelial cells. In the transcellular pathway, the absorption of bioactive substances mainly involves enterocytes and M cells (Figure 2). Enterocytes are specialised for transporting nutrients by active transport or passive diffusion [33]. Passive diffusion is limited to lipophilic bioactive substances with a molecular weight below 700 Da [34], while hydrophilic small molecules or lipophilic macromolecules are difficult to transport in this way. The apical membrane of enterocytes features a microvillus structure, which generates a large surface area that enhances the diffusion and absorption of nutrients and water from the intestinal lumen into the blood. Therefore, enterocytes are often used as targets for designing delivery platforms to maximise the delivery of bioactive substances to the small intestine, thereby increasing their bioavailability [15]. M cell-mediated intestinal lymphatic transport is an important pathway for absorbing lipophilic bioactive substances, which can bypass the liver, avoid first-pass metabolism and directly enter the systemic circulation via the mesenteric lymph [15]. The high transcytosis capacity (promoting efficient secretion of soluble substances and nanoparticles) and low endolysosomal activity (i.e., no intracellular digestion) of M cells are the bases for their use as targets for designing delivery platforms [35,36]. In addition, M cells can specifically participate in the absorption of microorganisms and antigens in the intestinal lumen [37]. Hence, targeting M cells is an effective means of vaccination [38].

The absorption of bioactive substances can occur not only by the transcellular pathway but also via the paracellular pathway of opening tight junctions between cells [39,40]. In particular, hydrophilic molecules prefer to be transported through the paracellular pathway [6]. Through this pathway, nanocarriers bypass the cell, thereby avoiding intracellular degradation [41]. Although bioactive substance-loaded carriers can also be transported and absorbed by the human body through this pathway, it may not be the best way to deliver bioactive substances as it only occupies less than 1% of the intestinal surface [42]. In addition, under normal conditions, tight junctions allow a size range of only 0.3–1.0 nm; even when entirely opened, their maximum value does not exceed 20 nm [43]. Paracellular transport by opening tight junctions may disrupt tight junctions, thereby increasing the risk of autoimmune diseases such as inflammatory bowel disease [44].

In the current study of targeting the small intestine, passive targeted nanodelivery systems are mainly designed using the characteristics related to the chemical barrier, biological barrier and mucus layer barrier in the physical barrier of the gastrointestinal tract, whereas active targeted nanodelivery systems are primarily constructed using the barrier properties of the intestinal epithelial layer in the physical barriers.

## 3. Passive Targeted Nanodelivery Systems

**Figure 3:**
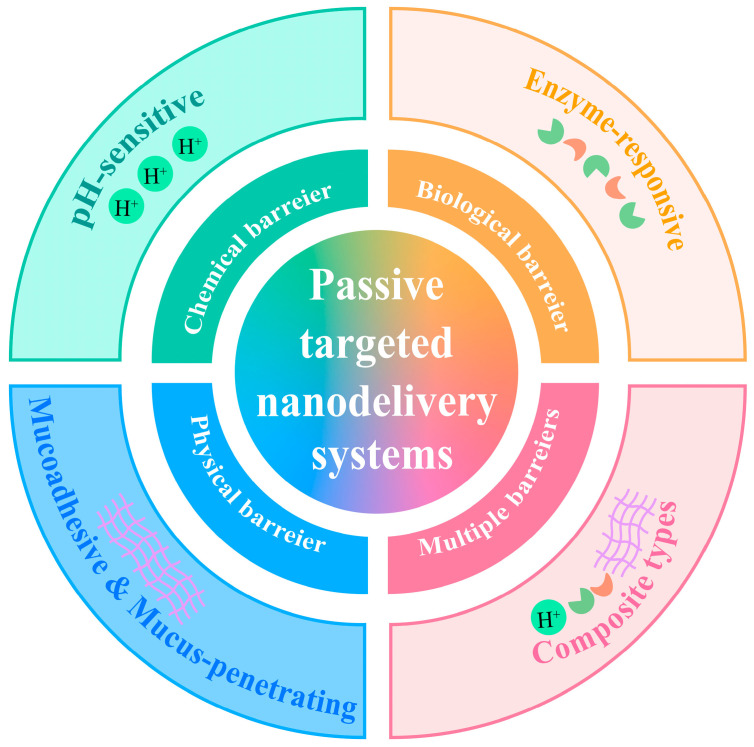
Passive targeted nanodelivery systems for different barriers.

Ideal delivery systems should transport bioactive substances along the gastrointestinal tract and protect them from damage by its harsh environment, thereby facilitating the absorption of bioactive substances in the small intestine [45]. In this part, passive targeted nanodelivery systems are mainly classified into pH-sensitive, enzyme-responsive, mucoadhesive, mucus-penetrating and composite types (Figure 3). Each type will be discussed individually in subsequent sections.

### 3.1. pH-Sensitive Nanodelivery Systems

Unlike the strongly acidic environment of the stomach, the pH of the small intestine is nearly neutral. Therefore, the pH difference between the small intestine and stomach can be utilised as a triggering factor for the release of bioactive substances to construct delivery systems [46]. The design of pH-sensitive nanocarriers is a promising strategy for controlled release. Such carriers have been shown to enhance the stability of bioactive substances in the stomach and enable their controlled release in the intestine [47]. Such delivery systems can solve the premature release of bioactive substances in the gastrointestinal tract and their insufficient accumulation in the target tissue (the small intestine). They are generally composed of a pH-responsive coating or matrix, which prevents the degradation of bioactive substances due to the disintegration of the carrier in gastric acid [48].

The most common pH-sensitive delivery systems are constructed by forming polyelectrolyte composite nanoparticles. They are formed by electrostatic interactions between two oppositely charged polyelectrolytes in aqueous solution [49]. This is a method for preparing nanoparticles without crosslinkers and homogenizers, which is beneficial to weaken the damage to the encapsulated components. As a result of changes in ionisation degree of functional groups, they will shrink, expand and even disintegrate in response to the pH changes in environmental solution [50]. To date, some pH-responsive nanodelivery systems have been developed by using polyanions and polycations to form polyelectrolyte composite nanoparticles. Considering that natural biopolymers have good biocompatibility, biodegradability and renewability, the pH-sensitive nanodelivery systems are mainly constructed based on the interaction between chitosan, the unique positively charged natural polysaccharide, or its derivatives and negatively charged natural polymers (such as alginate and pectin).

The pKa of the amino group in chitosan is about 6.5, which is protonated at low pH and deprotonated at pH > 7.0 [51]. The existence of this active functional group in chitosan provides favourable conditions for enhancing the gastric stability of bioactive substances and improving their permeability in the intestine, which can respond to pH changes from the acidic gastric region to the neutral intestine by protonation or deprotonation; therefore, it is suitable for constructing a pH-sensitive delivery system targeting the small intestine [52]. Common anionic groups including carboxyl groups (such as gum and alginate) and sulphate groups (such as carrageenan and fucoidan) also have similar properties, which can respond to changes in pH, thereby reducing the release of bioactive substances in the stomach and promoting their release in the intestine [53,54].The pH-sensitive nanodelivery systems based on the combination of chitosan or its derivatives with natural anionic polysaccharides have been successfully used in the study of targeted delivery of bioactive substances such as resveratrol [55], anthocyanins [56,57], curcumin [58,59,60], astaxanthin [61,62], oyster peptides [63] and ginsenoside [64,65] to the small intestine. Specifically, a previous study demonstrated that after dropping chitosan solution into solid lipid nanoparticle solution as a pH-responsive coating, the resulting mixture further formed capsosomes with liposomes, in which calcein showed obvious pH-responsive release characteristics [66]. In another study, a nanogel was successfully prepared by adding curcumin solution dropwise to a matrix solution composed of lotus root amylopectin and chitosan, followed by magnetic stirring [67]. The curcumin in the nanogel was released less than 16% in simulated gastric fluid for 2 h, and the cumulative release rate was about 70% after 4 h in simulated intestinal fluid. These nanodelivery systems enabled the above bioactive substances to be released in large quantities in a simulated small intestinal digestion environment or effectively improved the bioaccessibility of above bioactive substances in simulated digestion experiments, without the general use of animal models for bioavailability evaluation.

In addition to natural polymers, some synthetic polymers have been used to prepare pH-sensitive gastrointestinal nanodelivery systems. For example, hypromellose acetate succinate has been widely used as enteric coating material because it can remain intact at acidic environments and undergo deprotonation and dissolution at pH ≥ 6.5 [68]. Eudragit is a poly(methacrylic acid-methacrylate) copolymer that has also been extensively used in the formulation of pH-responsive delivery systems. Eudragit L100-55, Eudragit L100 and Eudragit S100 are pH-dependent anionic polymers, which can be dissolved in environments with pH values higher than 5.5, 6.0 and 7.0, respectively. Therefore, they can be used to construct nanodelivery systems targeting the duodenum, jejunum and ileum, respectively [69]. A recently released study found that the release rates of curcumin in curcumin nanosuspension, 4-layered modified (poly-L-arginine/sodium alginate)_2_ and Eudragit L100-coated stimuli-responsive nanosuspensions were 37.5%, 8.9% and 4.9%, respectively, in simulated gastric tract for 72 h but 2.2%, 8.9% and 11.7%, respectively, in simulated small intestinal tract for 72 h [70]. Nonetheless, pH-sensitive nanodelivery systems focus primarily on the impact of chemical barriers corresponding to pH on the delivery system, largely ignoring the effect of other gastrointestinal barriers.

### 3.2. Enzyme-Responsive Nanodelivery Systems

The environment of multiple enzymes present in the small intestine can be used to construct enzyme-responsive delivery systems. In terms of a single enzyme response, researchers used chitosan and adenosine triphosphate to self-assemble to form nanoscale spherical aggregates, which can be destroyed by the intestinal alkaline phosphatase (hydrolyzing adenosine triphosphate to single-negative-charge phosphates and a neutral adenosine) present in the brush border membrane of enterocytes [71]. Chitosan nanoparticles cross-linked with tripolyphosphate were also used to deliver β-galactosidase [72]. In this study, intestinal alkaline phosphatase can catalyse the hydrolysis of phosphate esters into inorganic phosphates and alcohol, resulting in the cleavage of the nanoparticles and the release of β-galactosidase. In another study, casein was applied as a coating for nanoparticles to enhance the stability of the delivery system in the acidic and enzymatic environment of the stomach, as well as to respond to trypsin in the small intestine and facilitate the absorption of hydrophobic actives in the small intestine [73].

To date, numerous studies have used the response of multiple digestive enzymes to construct nanodelivery systems. Among them, natural proteins or proteins and polysaccharides are the main materials for constructing nanocarriers. For example, the anthocyanin-loaded nanoemulsion formed by modified starch showed good stability in simulated gastric fluid, but was decomposed by amylase in simulated intestinal fluid [74]. Some amphiphilic proteins can self-assemble into nanoparticles for encapsulation and delivery of bioactive substances, enhancing their stability in the gastrointestinal tract [75,76]. For example, the nanoemulsion formed by amphiphilic soybean lipophilic protein enabled the targeted release of curcumin in the small intestine [77]. Similar results were obtained in the study of using casein to form nanoparticles to deliver apigenin [78]. The specific results of the above research can be found in Table 1. In addition, the nanotubes formed by the self-assembly of partially hydrolyzed α-lactalbumin released 90% of lycopene in simulated gastrointestinal digestion, and most lycopene was released in the small intestine [79].

However, compared with protein-based carriers that are susceptible to pepsin destruction, polysaccharide-based carriers are more stable and have greater resistance to harsh conditions such as extreme pH, ionic strength and temperature [83]. Therefore, to enhance the stability of single protein-based nanodelivery systems for the delivery of bioactive substances, polysaccharides are often combined with them. Furthermore, protein–polysaccharide complexes tend to have better functionality than individual proteins or polysaccharides [84,85].

In the nanodelivery systems formed by non-covalent interactions between proteins and polysaccharides, the interaction between charged polysaccharides and proteins is mainly electrostatic attraction, whereas that between neutral polysaccharides and proteins is mainly hydrogen bonding. A recent study showed that curcumin encapsulated in positively charged quaternary ammonium chitosan-coated zein–sodium caseinate nanoparticles had the highest bioavailability [80]. Similarly, the composite nanoparticles constructed by gliadin and sodium carboxymethyl cellulose also improved the stability and bioaccessibility of phloretin [82]. In the study of using sodium caseinate and konjac glucomannan (a neutral polysaccharide) to form nanoparticles for the delivery of mulberry anthocyanins, the nanoparticles released 27.25% in simulated gastric juice for 2 h, which was much lower than 54.47% of free anthocyanins [81]. The specific results of the above research can be found in Table 1. In addition, the composite nanoparticles formed by xanthan gum (an anionic polysaccharide) and lysozyme were also revealed to enhance the gastrointestinal stability of selenium-containing tetrapeptides and selenium-containing hexapeptides [86].

Besides non-covalent interactions, the combination of proteins and polysaccharides can also be carried out by covalent interactions. At present, the covalent binding of proteins and polysaccharides is generally carried out through the Maillard reaction, and the conjugates are used for the construction of nano-delivery systems. It is one of the effective methods to promote the conjugation of proteins and polysaccharides, which can improve the stability of protein without using toxic chemical reagents [87,88,89,90]. For example, the bioaccessibility of free curcumin was less than 20% after simulated digestion, but it was about 50% and 75% in nanoparticles based on oat protein isolate and oat protein isolate–*Pleurotus ostreatus* β-glucan conjugation, respectively [91]. A similar trend was observed in the study of β-carotene delivery using glycosylated Oat protein isolate nanoparticles [92]. Compared with non-covalent interactions, protein–polysaccharide conjugates formed by covalent interactions have stronger stability and other functional properties [93,94,95]. Therefore, ideally, the nanodelivery systems constructed by protein–polysaccharide covalent complexes are more advantageous in resisting the harsh environment of the stomach, thereby improving the bioaccessibility and bioavailability of bioactive substances [96].

Similarly to pH-sensitive nanodelivery systems, existing research on enzyme-responsive systems has predominantly substituted bioavailability assessments with bioaccessibility measurements in most published studies. To date, the gold-standard protocol for obtaining bioaccessibility data through in vitro simulated digestion remains the INFOGEST method [97]. Nevertheless, methodological concerns arise regarding the dialysis-based approaches employed in some investigations. Specifically, the molecular weight cutoff limitation of dialysis membranes prevents digestive enzymes in the external compartment from interacting with nano-delivery systems contained within the dialysis apparatus. Furthermore, both delivery systems fail to account for the obstruction posed by additional gastrointestinal barriers, particularly physical barriers governing the penetration and absorption mechanisms of bioactive substances.

### 3.3. Mucoadhesive Nanodelivery Systems

After overcoming the unfavourable effects of gastric acid or/and digestive enzymes, the next challenge for the nanodelivery system is the mucus layer that covers the absorptive epithelial cell layer of the intestine, which is one of the physical barriers. Mucoadhesive nanodelivery systems typically prolong the intestinal residence time by adhering to the intestinal mucus [98]. Near the intestinal wall, they allow the encapsulated bioactive substances to be continuously released at the target position and diffused into the mucus layer, thereby increasing the concentration of bioactive substances that can pass through the cell barrier and improving the delivery efficiency.

Hydrophobicity, surface charge and chemical properties are the three major factors affecting the mucoadhesive properties of polymers. Hydrophobicity and surface charge are mainly reflected by non-covalent interactions, whereas chemical properties are manifested in terms of covalent interactions.

With regard to non-covalent interactions, mucins can not only produce mucoadhesive properties by chain entanglement with substances [99] but also directly bind to hydrophobic substances through hydrophobic interactions [100]. The terminal hydrophobic region of mucins is usually considered to be the main reason for its interaction with hydrophobic substances [100]. In addition, targeting mucus can be achieved by exploiting the electrostatic interactions between the anionic charge of mucus and cationic polymers such as chitosan [101].

The mucoadhesive characteristics of cationic polymers are conducive to the delivery and absorption of encapsulated substances in the nanodelivery systems at specific sites [102]. For example, a study showed that cationic liposome containing dimethyl dioctadecyl ammonium bromide (a cationic ammonium salt) and conventional liposome without the ammonium salt only released 10%–12% of lysozyme in simulated gastric fluid for 4 h, while the conventional liposome only released 35% in simulated intestinal fluid for 8 h, and the cationic liposome released 53 [103]. In addition, the cationic liposome showed enhanced mucoadhesive properties upon co-incubation with porcine mucins, and the bacteriolytic activity of lysozyme-loaded cationic liposome after simulated digestion was much higher than that of lysozyme alone and the conventional liposome.

Anionic polymers, especially polysaccharides such as alginate [104], hyaluronic acid [105] and carboxymethyl cellulose [106] can also be used to construct mucoadhesive delivery systems. Anionic polymers, such as some polysaccharides with carboxyl groups, carry a negative charge at neutral pH, and their strong mucoadhesive properties may be related to hydrogen bonding and van der Waals forces [107]. However, mucoadhesive nanodelivery systems constructed only by anionic polymers may produce electrostatic repulsion with negatively charged mucins to make them not dominant in adhesion, so the related research is still relatively limited.

On the one hand, the mucoadhesive properties are based on the above non-covalent bonds, including chain entanglements, hydrophobic interactions, electrostatic interactions, hydrogen bonds and van der Waals forces; on the other hand, this performance is based on the covalent bonds between the active functional groups of polymers and the substructure of cysteine in mucus glycoprotein [26,108]. These non-covalent forces are weaker than the covalent bonds formed between polymers and the mucus layer. To obtain the ability to form covalent bonds with mucins, polymers should be coupled with substances containing thiol, catechol, boronate, acrylate, methacrylate, maleimide, and N-hydroxy(sulpho)succinimide ester groups [109]. Among them, introducing thiol groups into water-soluble polymers has become the most widely explored strategy for enhancing mucoadhesive properties.

Furthermore, the formation of mucoadhesive thiolated polymers through covalent disulphide bonds can be cationic, anionic or even neutral. For cationic polymers, thiolated chitosan can adhere tightly and anchor in the mucus layer to prolong the residence time, which is formed by the electrostatic interactions between chitosan and mucin, as well as the covalent disulphide bonds formed by the free thiol groups and the mucus cysteine domain [110]. For anionic polymers, Laffleur et al. [111] achieved thiolation modification by forming amide bonds between the carboxyl groups of four anionic polysaccharides (carboxymethyl cellulose, hyaluronic acid, polycarbophil and alginate) and the primary amino groups of L-cysteine. Their results showed that the adhesion properties of all polysaccharides were significantly improved after modification, and the total adhesion work after modification was 2.18, 2.77, 16.29 and 59.23 times that before modification, respectively. For neutral polymers, the per-thiolated β-cyclodextrin/coumarin-6 host-guest complex was also demonstrated to be 19.4, 2.1 and 4.5 times more abundant than the native β-cyclodextrin group in the stomach, duodenum and jejunum and ileum of rats, respectively [112].

Nevertheless, mucoadhesive nanodelivery systems also have some limitations. They may adhere non-specifically to surfaces to which they are not intended to adhere (e.g., gastric mucosa and intestinal contents) [113]. In addition, the loose mucus layer present in the small intestine is constantly being renewed. The mucoadhesive nanodelivery systems trapped in loose mucus layer may be removed with the renewal of mucus, reducing the absorption of bioactive substances by the human body. Therefore, it is critical to avoid excessive adhesion when designing mucoadhesive nanodelivery systems [114].

### 3.4. Mucus-Penetrating Nanodelivery Systems

Another factor limiting delivery efficiency is how to quickly penetrate the mucus layer to reach the following intestinal cells. Compared with mucoadhesive nanodelivery systems, mucus-penetrating nanodelivery systems may be a more promising strategy to increase the absorption of bioactive substances. They can improve the distribution and permeability of the delivery platform in the mucus layer so that they can reach the epithelial cells and subsequently be absorbed. To effectively penetrate the mucus layer, the delivery carrier needs to reduce contact with it. Reducing interactions such as hydrophobic or electrostatic interactions between the delivery system and mucus can effectively reduce their adhesion. Mucus-penetrating nanodelivery systems can typically be achieved by endowing them with hydrophilic and uncharged surface properties similar to those of viruses that can move quickly in human mucus [115].

Polyethylene glycol (PEG) is a non-ionic and net-neutrally hydrophilic polymer [116]. Coating nanoparticles with high-density, low-molecular weight PEG has been shown to reduce the interaction between particles and mucus [115]. In recent years, coating nanoparticles with low-molecular weight PEG is one of the common methods for manufacturing mucus-penetrating systems. The attachment of PEG to the surface of nanocarriers is commonly referred to as PEGylation, which has been the gold standard strategy for resisting non-specific protein adsorption. PEG has favourable safety and physicochemical properties and has traditionally been regarded as biologically inert and non-immunogenic [117]. However, clinical studies have shown that anti-PEG antibodies have also been detected in the blood of some healthy individuals who have never been exposed to PEG-containing drugs [118,119]. PEG is frequently found in many foods and oral and topical products, which may elevate levels of PEG-specific antibodies in the whole body and/or mucosa over time, thereby inducing associated anti-PEG immunity [120]. Anti-PEG antibodies can effectively capture PEG-modified particles, block them in the mucus and significantly reduce their diffusion. A recent study revealed that liposomes with low-density PEG are still susceptible to destabilisation by anti-PEG antibodies [121]. In addition, although high-density PEG modification can effectively avoid the interaction between various delivery systems and mucins, its high biological inertness weakens cellular uptake and endosomal escape properties [122]. A similar inert substance for mucus-penetrating systems is the amphiphilic copolymer Pluronic F127, which has a central hydrophobic poly(propylene oxide) segment and two terminal hydrophilic PEG portions [123,124]. However, not all substances with hydrophilic and neutral charges can be used for mucus penetration. For example, polyvinyl alcohol is a water-soluble non-ionic synthetic polymer, but it is traditionally classified as a mucoadhesive polymer [125]. However, an investigation has shown that non-covalently coating mucosal adhesive nanoparticles with partially hydrolysed polyvinyl alcohol (especially those with a degree of hydrolysis of <95% and at least <75%) can help nanoparticles migrate in mucus [126].

A research demonstrated that viruses with high concentrations of positively and negatively charged surface properties can diffuse as fast in mucus as in saline solution [127]. Net-neutral zwitterionic polymers consisting of both cationic and anionic groups usually have stronger hydrophilicity than PEG. In addition, their antifouling ability resists the adsorption of non-specific biological macromolecules (such as mucin) [128]. The strong hydration induced by electrostatic interaction is the key to the antifouling properties of zwitterionic materials [129,130], while the antifouling ability of PEG is attributed to the hydrogen bond with water [131]. Currently, zwitterionic polymers based on carboxybetaine, sulfobetaine and phosphocholine have been widely used to construct nanocarriers. Moreover, compared with PEG, zwitterions do not have corresponding specific antibodies and do not induce adverse immune responses. Shan et al. [132] prepared dilauroylphosphatidylcholine (a zwitterion)-functionalised nanoparticles. Compared with Pluronic F127- or polyvinyl alcohol-modified nanoparticles, the zwitterionic functionalised nanoparticles have the mucus-penetrating ability of F127-functionalised nanoparticles and the cellular uptake ability of polyvinyl alcohol-functionalised nanoparticles. Moreover, zwitterionic residues have been reported to be involved in cellular uptake mediated by proton-assisted amino acid transporter 1 (PAT1) in the small intestine [133]. These results demonstrate that zwitterionic polymers have better application potential in mucus-penetrating nanodelivery systems than substances such as PEG and Pluronic F127 that do not have high-density charge.

In addition to directly preparing zwitterions and bioactive substances into nanoparticles/micelles for delivery, zwitterions can also be used as a functional coating of nanodelivery particles, thereby conferring the composite nanoparticles mucus-penetrating function, improving the bioavailability of bioactive substances. Chen’s team achieved efficient loading of exenatide through zwitterionic hydrogel-coated metal–organic framework (MOF) nanoparticles [134]. The zwitterionic hydrogel layer significantly improved the ability of MOF nanoparticles to penetrate intestinal mucus and promoted the uptake of intestinal cells, which was ultimately beneficial to the absorption and utilisation of exenatide in the intestine. Moreover, in a rat model of diabetic, the zwitterionic hydrogel-coated nanoparticles encapsulated in enteric capsules significantly increased the utilisation efficiency of exenatide and reduced the blood glucose concentration in rats.

Even though zwitterionic modification can enhance the endocytosis of nanocarriers, it usually cannot increase the desired transcytosis. Therefore, nanocarriers normally have the problem of ‘easy entry hard transcytosis’. The polyzwitterion poly(2-(*N*-oxide-*N*,*N*-diethylamino)ethyl methacrylate) (OPDEA)-based delivery system has been found to facilitate the rapid uptake and transcytosis of tumour endothelial cells and cancer cells [135]. The research team used OPDEA, which is highly non-fouling to proteins and other biological macromolecules (such as mucin) but can weakly bind to phospholipids, to design a block copolymer with poly(ε-caprolactone) (OPDEA–PCL) to form nanomicelles for paclitaxel delivery [136]. The nanomicelles can effectively penetrate the mucus layer and bind to villi, triggering transcytosis-mediated transepithelial transport (the non-lysosomal pathway) into the blood circulation for tumour accumulation. The bioavailability of the micelles was 28.73%. whereas those of the PEG–PCL nanomicelles and free PTX were only 6.22% and 2.43%, respectively.

However, to date, all zwitterionic polymers based on radical polymerisation of vinyl betaines have the main chains composed of noncleavable carbon-carbon bonds, so the accumulation of these non-degradable polymers in the body may have adverse effects on the body [137]. Although betaine-decorated biodegradable materials are completely biodegradable, the presence of non-zwitterionic segments may decrease the concentration of zwitterionic groups in the polymer, thereby impairing the properties of zwitterions [138].

Therefore, in zwitterionic-modified biodegradable materials, compared with synthetic materials, the development of natural materials must be increased. Proteins with appeared as natural amphoteric electrolytes may be potential materials for constructing nanocarriers to overcome the mucus layer. Zhao’s group has advanced the study of using soy protein isolate-based mucus-penetrating nanoparticles for curcumin delivery [139,140]. However, these studies failed to validate the gastrointestinal stability of the nanodelivery systems, particularly their structural integrity maintenance during digestion, a critical prerequisite for ensuring mucus-penetrating capability. Furthermore, when constructing a mucus-penetrating delivery system, a balance should be found between the zwitterionic concentration and the antifouling and mucus penetration properties of the final system.

### 3.5. Composite Nanodelivery Systems

In passive targeted nanodelivery systems, compared with a single type of delivery system, composite nanodelivery systems that consider multiple barriers have been shown to be more advantageous in the delivery of bioactive substances. This part will mainly introduce the nanodelivery systems based on pH-sensitive and mucoadhesive, mucoadhesive and mucus-penetrating and enzyme-responsive and mucus-penetrating properties.

In pH-responsive and mucoadhesive composite nanodelivery systems, the nanodelivery systems for insulin delivery prepared from hyaluronic acid with different molecular weights and poly[2-(dimethylamino)ethyl methacrylate] exhibited effective hypoglycaemic effects and relative bioavailability of up to 14.62% in pharmacodynamic and pharmacokinetic studies in rats, respectively, while free insulin had no significant hypoglycemic effect and relative bioavailability was only 0.66% [141]. In another study, researchers developed a nanodelivery system (pH-TCS/O-CMCS @ LMWH) with a particle size of about 332 nm for the delivery of low-molecular weight heparin (LMWH) using thiolated chitosan (TCS) and o-carboxymethyl chitosan (O-CMCS) [142]. Its bioavailability in rats was 34.53%, which was 17.2, 1.66 and 2.14 times that of LMWH solution, pH-TCS@LMWH and pH-CS/O-CMCS@LMWH groups, respectively.

In mucoadhesive and mucus-penetrating composite nanodelivery systems, researchers synthesised hydrophobic palmitic acid and cysteine-modified N-2-hydroxypropyl trimethyl ammonium chloride chitosan to form an amphiphilic polymer, and then used it to prepare curcumin-loaded nanomicelles [143]. The bioavailability of the nanomicelles was 6.42 and 2.45 times that of the free curcumin and without cysteine-modified groups, respectively. In this study, electrostatic interactions and disulfide bonds were the main reasons for mucoadhesion. However, the formation of disulfide bonds also weakened the interaction between mucins, “diluting” mucus and facilitating the penetration of the nanoparticles [144]. In another study, researchers fabricated lipid-polymeric nanoparticles (SLPNs) coated with amphiphilic sophorolipid assemblies (with the ability to resist high salt concentration and acidic conditions) by nanoprecipitation and self-assembly [145]. SLPNs composed of polylactide-co-glycolide (PLGA, a biodegradable polymer [146]) and sophorolipid nanocore, with a phospholipid inner shell and sophorolipid assembly outer shell, was electrically neutral and had good stability in simulated gastrointestinal fluid. The strong affinity of the outer shell of SLPN to mucins led to its dissociation during mucus penetration, and the remaining part rapidly penetrated the mucus layer in a biologically responsive manner. Moreover, the results of pharmacokinetic study in rats showed that compared with silibinin suspension, SLPN co-loaded with silibinin (a flavonoid substance) and curcumin increased the maximum plasma concentration and bioavailability of silibinin by 32.31 and 11.48 times, respectively, while these two values were 16.30 and 12.00 times in nanoparticles without a sophorolipid outer layer, respectively; the maximum plasma concentration and bioavailability of curcumin in SLPN were 23.09 and 11.9 times that of the curcumin suspension, respectively, and 3.25 and 1.58 times that of nanoparticles without a sophorolipid outer layer.

In enzyme-responsive and mucus-penetrating composite nanodelivery systems, researchers anchored the cationic octa-arginine (R8) peptide, a cell-penetrating peptide and anionic phosphoserine (Pho), which is a substrate of intestinal alkaline phosphatase on the surface of PLGA to obtain virus-like densely charged but neutral surface, nanoparticles (P–R8–Pho) [147]. P–R8–Pho can reach the intestinal epithelium after penetrating the mucus layer, so Pho is hydrolyzed by intestinal alkaline phosphatase to expose R8, thereby mediating efficient cellular uptake and transepithelial transport. The composite nanodelivery system solved the conflict properties between hydrophilic and neutral surface that promote mucus penetration and hydrophobic and cationic surface that promote intestinal epithelial cell uptake, which exists in a single nanocarrier. In this study, the bioavailability of free insulin was only 0.4%, and the bioavailability of insulin in P–R8–Pho (5.96%) was 2.3 and 1.9 times that of nanoparticles without R8 and Pho decoration, and nanoparticles only modified by R8, respectively.

In terms of passive targeted nanodelivery systems, compared with single types, composite delivery systems have better application prospects in promoting the absorption of bioactive substances, as they take into account more gastrointestinal absorption barriers. Nevertheless, they still ignore the last barrier to overcome the absorption to a certain extent, namely, the intestinal epithelial cell layer.

The advantages and disadvantages of each passive targeted delivery system can be viewed in Table 2.

## 4. Active Targeted Nanodelivery Systems

Compared with the passive targeted nanodelivery system, the active targeted nanodelivery system is designed for a smaller cellular level and is mainly based on ligand–receptor/transporter-mediated transport. The active targeting of cells can be achieved by the interaction between the ligands on the delivery system and the receptors on the cell surface, thereby enhancing the enrichment of bioactive substances at the target site and mitigating their off-target effects. Transporters are also important membrane transport proteins involved in the transport of nutrients and other substances [148]. Therefore, they are an effective way to improve the cellular uptake of bioactive substances and increase their bioavailability by attaching ligands that can specifically recognise cell surface receptors/transporters to the surface of nanodelivery systems.

**Figure 4:**
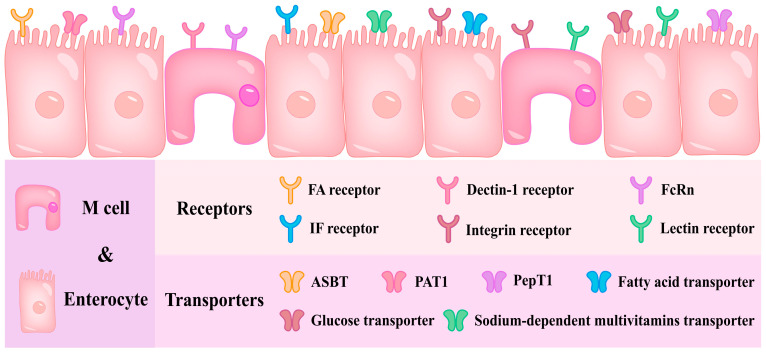
Some receptors and transporters exist in M cells and enterocytes for the design of nanodelivery systems.

However, the uptake of intact nanocarriers is a prerequisite for active targeted delivery mediated by ligand–receptor/transporter transport [149], that is, nanocarriers need to overcome the chemical barrier, biological barrier and mucus layer barrier in the gastrointestinal tract to ensure their intact morphology before reaching epithelial cells. Active targeted nanodelivery systems generally have the characteristics of mucus-penetrating nanodelivery systems. The transcellular pathway based on active targeted delivery is generally carried out by targeting M cells or enterocytes [150]. A variety of receptors expressed on the surface of M cells and multiple receptors/transporters expressed on the surface of enterocytes can be used to design targeted nanodelivery systems [151]. Figure 4 shows some receptors and transporters for the design of nanodelivery systems.

In addition, nanocarriers may not be able to achieve ‘lysosomal escape’ during transport into the systemic circulation by enterocytes, which are the main intestinal barrier cells. In other words, the nanocarriers may be digested and decomposed by the lysosome in enterocytes and inactivate the loaded bioactive substances, decreasing their bioavailability. By contrast, M cells, which make up only a small proportion of the intestinal epithelial cells, can effectively transport a variety of macromolecules or particles from the intestinal lumen to the Peyer’s patch without digestion [152]. However, delivery systems can interact with basolateral immune cells (such as dendritic cells and T cells) after passing through M cells, which usually prevents the delivery system from entering the systemic circulation [149].

However, current research on active targeting at the cellular level predominantly concentrates on applications in drug delivery. In contrast, this approach is still in its early stages within the fields of food and nutrition. Consequently, we aim to leverage insights from advancements in drug delivery systems to guide our research strategies for the targeted delivery of food-derived bioactive substances.

### 4.1. Single Ligand–Receptor-Mediated Nanodelivery Systems

Neonatal Fc receptor (FcRn) is a Fc receptor that binds immunoglobulin G via electrostatic interactions at acidic pH and mediates the transcytosis pathway through the intestinal mucosal barrier [153]. It always exists in the intestines of newborns and adults. Therefore, the bioactive substance-loaded delivery system with FcRn targeting ability can effectively overcome the intestinal epithelial barrier and improve their bioavailability [154,155]. In a recent study, researchers used a maleimide–thiol chemical method to couple FcRn targeting peptide and FcRn affibody molecule on PLGA–PEG, respectively, to form nanoparticles for targeting intestinal cells [153]. Compared with non-targeted nanoparticles, both targeted nanoparticles exhibited a twofold increase in interactions with intestinal cells that endogenously express human FcRn. The FcRn targeting ability of the targeted nanoparticles was also confirmed in human intestinal organoids, a 3D in vitro intestinal model, that expresses human FcRn.

The binding mode of vitamin B12 (VB12) to intestinal epithelial cells is different from that of general ligands, because it does not work through direct binding between itself and the receptor. VB12 first binds to an intrinsic factor (IF) produced by the stomach to form a VB12–IF complex, which further binds to the IF receptor on the surface of intestinal epithelial cells and is then transported to circulation [156]. Although the receptor for the VB12–IF complex is expressed on enterocytes in the duodenum, jejunum and ileum, the majority is located in the ileum; the IF of the complex is degraded during transport through the lysosomal pathway [30]. In addition, VB12-modified nanoparticles are thought to be able to be transported in intestinal epithelial cells via the lysosomal escape pathway [157]. In a study that used VB12-modified amphiphilic chitosan to form nanomicelles with scutellarin via hydrophobic interactions, the bioavailability of the targeted nanomicelles in rats (22.10%) was 3.44 and 1.70 times higher than that of free scutellarin and untargeted modified nanomicelles, respectively [158].

In addition to considering the targeting of the nanodelivery systems to the intestine, the nanodelivery systems with ‘secondary targeting’ ability to re-target the target organ/tissue after targeting the intestinal epithelial cells into the systemic circulation will be promising in improving the bioavailability of bioactive substances. The results of using dextran–bovine serum albumin conjugate mixed with protamine as an emulsifier to form 1,8-cineole-loaded nanoemulsions and further modified with VB12 showed that the VB12-modified nanoemulsions could effectively overcome various barriers of the gastrointestinal tract [159]. Furthermore, they can be targeted to atherosclerotic plaques after entering the blood due to the presence of dextran, and the bioavailability of 1.8-cineole-loaded by them in mice is 13.5 and 3.3 times higher than that of free 1.8-cineole and VB12-unmodified nanoemulsions, respectively.

Similarly, researchers have used the ability of β-glucan to target the dectin-1 receptor of M cells and macrophages and then participate in blood circulation through hitchhiking macrophages to construct nanoparticles for lutein delivery by using phycocyanin, chitosan and 3-boronobenzoic acid-modified yeast β-glucan yeast [160]. The lutein-loaded nanoparticles enhanced the stability of lutein in the gastrointestinal tract and improved its absorption efficiency in the small intestine. The concentration of lutein in nanoparticles in plasma and eyeballs was 2.63 and 1.81 times higher than that of free lutein, respectively. The nanoparticles can effectively prevent the occurrence of dry eye disease in a mice model. However, it should be noted that this study did not explore the capacity of nanoparticles to penetrate the intestinal mucus layer, nor did it examine the integrity of these particles following simulated digestion. Maintaining particle integrity is essential for their effective targeting of intestinal epithelial cells.

Some studies based on single ligand–receptor-mediated targeted nanodelivery systems are listed in Table 3, such as folic acid (FA) receptors, FcRn. According to the results listed in Table 3, the bioavailability of bioactive substances in nanocarriers via the ligand–receptor-mediated absorptio2 pathway was significantly higher than that of free bioactive substances and nanocarriers without ligand modification. Additionally, some ligand–receptor transport pathways in intestinal epithelial cells that cannot mediate the ‘lysosomal escape’ pathway or non-lysosomal pathway may reduce the final bioavailability of bioactive substances.

### 4.2. Single Ligand–Transporter-Mediated Nanodelivery Systems

Ligand–transporter-based nano-delivery systems are generally designed for enterocytes. In-depth understanding of the various transporters and modification of their corresponding ligands onto the surface of the nanocarriers can achieve intestinal targeting and enhance the absorption of the bioactive substances, thereby improving the bioavailability of bioactive substances. Some studies based on single ligand–transporter-mediated targeted nanodelivery systems are listed in Table 4, such as apical sodium-dependent bile acid transporter (ASBT), proton-dependent intestinal peptide transporter 1 (PepT1) and lipid transporter. Similarly to the ligand–receptor-mediated absorption pathway, the bioavailability of bioactive substances in nanocarriers modified by the targeted ligand via the ligand–receptor-mediated absorption pathway was significantly higher than that of free bioactive substances and unligand-modified nanocarriers.

Constructing a nanodelivery system capable of targeting lipid transporters for delivering carotenoids has promising prospects [173]. In a recent study, self-assembly of glycosylated zein with different types of fatty acids (such as myristic acid, palmitic acid and stearic acid) for the delivery of fucoxanthin (a carotenoid) showed that the nanodelivery system containing fatty acids had a bioaccessibility of up to 77.77% in simulated digestion experiments, whereas the nanodelivery system without fatty acids and free fucoxanthin solution were only 43.09% and 17.08%, respectively [174]. In addition, fatty acid-modified nanoparticles in this study used lipid transporters to promote the absorption of fucoxanthin in mice. Among them, palmitic acid-modified nanoparticles had the highest concentration of fucoxanthin metabolites in serum, which was 1.6 and 1.8 times higher than that of fatty acid-free nanoparticles and free fucoxanthin, respectively. Notably, the nanoparticles in this study could not maintain good stability in the gastrointestinal tract and did not involve mucus penetration studies. Therefore, the improved bioavailability and the increased expression of lipid transporters by fatty acids may be the reasons for the increased bioavailability.

Generally, the bioavailability of macromolecular proteins through the gastrointestinal tract is very poor (<1%) [175]. It has been reported that zwitterionic micelles formed by conjugating zwitterionic betaine polymers with 1,2-distearoyl-*sn*-glycero-3-phosphoethanolamine 1,2-distearoyl-sn-glycerol-3-phosphate ethanolamin can be transported across epithelial cells through the PAT1 pathway without opening tight junctions [176]. The bioavailability of insulin was as high as 42.6% when freeze-dried powder of zwitterionic micelle insulin was encapsulated in enteric capsules. Similarly, Fang et al. [169] used enteric capsules to load polyzwitterion/protein nanocomplexes formed by in situ polymerisation, allowing the nanocomplexes to be released in the intestine without being affected by the gastric environment. After that, they can penetrate the mucus layer and overcome the cellular barrier through the PAT1 pathway to enter the blood circulation. In this study, although all groups used enteric capsules, the bioavailability in the capsule group containing free insulin was only 1.3% in rats. In contrast, the bioavailability of polyzwitterion/insulin nanocomplexes and polyzwitterion/immunoglobin G nanocomplexes was as high as 16.9% in rats and 12.5% in mice, respectively.

In addition, ASBT, which is associated with bile acid transport, is highly expressed in the small intestine and has been used to develop active targeted nanocarriers. ASBT is mainly expressed on the apical region of ileal enterocytes, and it is highly expressed in the distal ileum, which contributes to the hepatoenteral circulation of bile acids. In a study, researchers developed deoxycholic acid-modified nanoparticles with endosomal escape function, which significantly prevented insulin degradation in intestinal epithelial cells [177]. The nanoparticles have been shown to interact with cytoplasmic ileal bile acid-binding proteins to promote intracellular transport and basolateral release of insulin. Pharmacokinetic studies showed that the bioavailability of freeze-dried deoxycholic acid-modified nanoparticles loaded into enteric capsules was 15.9% in diabetic rats after 12 h of administration, which was about 12.2 and 2.2 times higher than that of insulin alone and unmodified nanoparticles, respectively. This study used enteric capsules to help overcome gastrointestinal digestion, indicating that the nanoparticles themselves may have poor resistance to digestion.

In another study, researchers used 9-fluorenylmethoxycarbonyl-polyethylene–glycocholic acid to form high-density PEGylation-based glycocholic acid-decorated nanomicelles [178]. The flexible PEG of the nanomicelles conferred them the ability to penetrate mucus, and the presence of glycocholic acid with high affinity to ASBT enabled them to target intestinal epithelium. In vitro and in vivo results indicated that the oral nanomicelles could improve the bioavailability of paclitaxel. Especially in the pharmacokinetic study of rats, the targeted nanomicelles showed a high bioavailability of 21.2%, which was 7.43 and 1.69 times higher than that of free paclitaxel and untargeted nanomicelles, respectively.

Other studies have discussed ‘secondary targeting’ in single ligand–transporter-mediated nanodelivery systems. Recently, researchers modified the PLGA–PEG skeleton with mannose to target glucose transporter 1, which is highly expressed in intestinal epithelial cells and brain endothelial cells, and constructed a fingolimod-loaded nanodelivery system that can overcome both the intestinal epithelial barrier and the blood–brain barrier [179]. After 6 h of administration to mice, under a blood glucose control strategy, the accumulation of fingolimod in the brain of mice in this nanodelivery system increased to 13.2 times that of free fingolimod, and it had strong retention ability in the intestine. Nevertheless, whether this nanodelivery system can maintain its intact structure and enter systemic circulation after passing through the intestinal epithelial barrier remains unverified, as structural integrity is an essential prerequisite for achieving ‘secondary targeting’.

In addition to the environment of healthy humans, the high expression of specific transporters in certain pathological conditions can be used to design nanodelivery systems. For example, the upregulated expression of proton-coupled folate transporter in intestinal epithelial cells of diabetes can mediate the endocytosis and intracellular transport pathways (lysosome escape and Golgi transport) of folate-grafted nanoparticles, improving the bioavailability of insulin [148]. In the diabetic rat model, the bioavailability of enteric capsules loaded with folic acid-grafted nanoparticles was 14.4%, whereas that of enteric capsules containing nanoparticles without folic acid-grafting was only 5.2%. In addition, the bioavailability of enteric capsules loaded with folic acid-grafted nanoparticles decreased to 9.2% in normal rats. Notably, the study employed enteric capsules and ignored critical evaluation of nanoparticle-mediated mucus penetration efficacy. Despite these limitations, the findings offer valuable insights into precision nutrient delivery for metabolic dysregulation scenarios, particularly obesity-related comorbidities and subclinical malnutrition states.

Similarly to ligand–receptor-mediated active targeted nanodelivery systems, the current research on ligand–transporter-mediated nanodelivery systems still needs to further break through the dilemma of intestinal epithelial digestion, to effectively improve the bioavailability of bioactive substances. Furthermore, in many published studies, enteric capsules have been used to encapsulate nanocarriers to help them overcome the chemical and biological barriers of the gastrointestinal tract, so nanocarriers still have some limitations.

### 4.3. Multiple Ligand–Receptor/Transporter-Mediated Nanodelivery Systems

Compared with the single ligand–receptor/transporter-mediated active targeted nanodelivery system, the delivery system modified with multiple ligands may have better effects in improving the bioavailability of bioactive substances [180].

Xi et al. [181] constructed a functional nanoparticle (PG–FAPEP) co-modified with FA and charge-convertible tripeptide. Nearly neutrally charged PG–FAPEP could be internalised into intestinal epithelial cells through the FA-mediated ligand–receptor transport pathway and became positively charged due to the presence of the tripeptide when reaching acidic lysosomes, triggering the proton sponge effect for lysosomal escape. Thereafter, PG–FAPEP entering the cytoplasm was converted into electrical neutrality again, weakening intracellular adhesion and recognising the proton-coupled oligopeptide transporter (PHT1) in the basolateral membrane with the help of tripeptides, thereby promoting the intact exocytosis of PG–FAPEP in intestinal epithelium. The dual-modified PG–FAPEP encapsulated in enteric capsule showed a high bioavailability of 14.3%, which was 3.18, 2.13, 1.63 and 8.41 times that of the unmodified, FA-modified, tripeptide-modified nanoparticles and free insulin, respectively. This study demonstrated that nanocarriers can synergistically pass through the intestinal epithelial barrier via multiple ligand–receptor/transporter-mediated transport pathways to achieve the same function as a single ligand–receptor/transporter-mediated transport pathway that can perform lysosomal escape.

More recently, composite nanoparticles co-modified with zwitterion that mediate the PAT1 transport pathway and ursodeoxycholic acid that mediate the ASBT transport pathway have been used for co-delivery of insulin and berberine [182]. Insulin and berberine in the double-modified nanoparticles had the largest plasma concentration and bioavailability in diabetic mice. Moreover, given the co-modification of zwitterion and ursodeoxycholic acid to overcome multiple intestinal barriers, and the synergistic effect of insulin and berberine on hyperglycaemia, the composite nanoparticles exhibited the highest hypoglycaemic ability (21.2%), but it was only 18.3% and 14.2% in nanoparticles without ursodeoxycholic acid modification and nanoparticles without co-loaded berberine, respectively. This study solved the problem of ‘easy entry and difficult penetration’, that is, nanoparticles could be endocytosed by PAT1 but could not be effectively transcytosed in the intestinal epithelial barrier, which effectively improved the bioavailability of bioactive substances and enhanced the final effect.

## 5. Forms and Efficiencies of Passive/Active Targeted Nanodelivery Systems Entering Systemic Circulation

**Figure 5:**
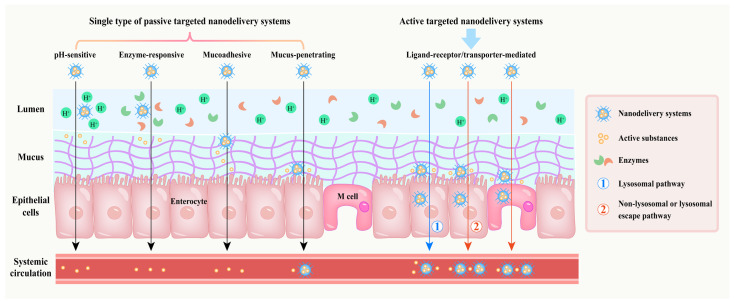
Different forms and efficiencies of passive and active targeted nanodelivery systems loaded with bioactive substances entering systemic circulation after overcoming the gastrointestinal barrier.

The existing studies on nanodelivery systems targeting for the absorption of bioactive substances have mainly focused on passive targeted nanodelivery systems, including pH-sensitive, enzyme-responsive, mucoadhesive, mucus-penetrating and composite nanodelivery systems. However, in addition to the nanodelivery systems with mucus-penetrating properties, other passive targeted delivery systems loaded with bioactive substances are not eventually absorbed by the intestinal epithelial cells in the form of intact carriers (Figure 5). Furthermore, the physiological barriers of the digestive tract are not fully considered in passive targeted nanodelivery systems, which limits the final bioavailability of bioactive substances.

The most ideal nanodelivery system should be able to protect the bioactive substances from the harsh gastrointestinal conditions, so as to target the specific receptor/transporter in an intact form to increase the uptake of intestinal epithelial cells, and mediate the intracellular transport and exocytosis of the nanodelivery system through the non-lysosomal pathway or lysosomal escape pathway. This phenomenon belongs to the active targeted nanodelivery system mentioned in this review (Figure 5). In addition, M cells account for a small proportion of the total surface area of the small intestine, so the most promising way to increase the bioavailability of bioactive substances through the intestinal wall will be by targeting the largest number of enterocytes with microvilli structure in the intestine.

## 6. Clinical Trials

Currently, nanodelivery systems targeting the small intestine face significant challenges in transitioning from laboratory settings to clinical applications, with only a small number of products advancing to the clinical trial stage [14]. For example, silica nanoparticles delivering insulin orally for the treatment of type I diabetes (National Clinical Trial No. NCT01973920) are currently in Phase II clinical trials [14]. It should be noted that species differences between animal models and humans often compromise the accuracy of predicting treatment outcomes in clinical trials. Additionally, many nanodelivery systems have not been comprehensively assessed for systemic and local toxicity in normal tissues. These issues are the reason for the slow progress in clinical research [183]. Although targeted delivery of bioactive substances to remote areas beyond the gastrointestinal tract is still in its early conceptual stages, the encouraging results achieved so far herald promising translational potential. [149].

## 7. Conclusions and Perspectives

The complex physiological barriers in the digestive tract prevent the body from absorbing and utilising bioactive substances. In this review, we describe the gastrointestinal barriers that bioactive substances need to pass through before they are absorbed by the human body and their characteristics. We further elaborate on how to construct targeted nanodelivery systems that can overcome different physiological barriers. We believe that constructing a nanodelivery system capable of actively targeting enterocytes will represent the optimal strategy for enhancing the bioavailability of bioactive substances.

Considering the factors of receptors/transporters, carrier materials, bioactive substances and biosecurity, the active targeted nanodelivery system constructed in the future should have the following characteristics: (i) using natural materials such as polysaccharides and proteins; (ii) does not rely on enteric capsules to overcome the gastrointestinal barriers; and (iii) can be transported and exocytosed by enterocytes in an intact form. Furthermore, researchers must develop co-delivery of bioactive substances with synergistic effects, especially in the co-delivery of hydrophilic and hydrophobic substances, as well as large and small molecular substances. On the basis of these foundations, active targeted nanodelivery systems with ‘secondary targeting’ capabilities have strong development potential, because they can be further enriched in targeted organs/tissues (such as kidney, eye and adipose tissue) after entering the blood to intensify the effect of bioactive substances. Although there are currently efforts to construct targeted nanodelivery systems by leveraging differences in intestinal epithelium between healthy and certain disease states (such as diabetes), most research is still based on the gastrointestinal environment under healthy conditions. As a consequence, further attention needs to be paid to the differences in the complex gastrointestinal environment between healthy and sub-healthy states, as well as some diseases that have not yet been studied, to design differentiated delivery strategies and promote the development of fields such as personalised nutrition and nutritional intervention.

## Figures and Tables

**Table 1:** Some enzyme-responsive nanodelivery systems.

Bioactive Substances	Main Material	Model	Main Results	References
Anthocyanin	Modified starch	In vitro and vivo	The bioavailability of anthocyanin in nanoemulsions in mice was about 2 times that of free anthocyanin.	[74]
Curcumin	Soybean lipophilic protein	In vitro and vivo	The nanoemulsion increased the bioaccessibility of curcumin from 46.33% of free curcumin to 76.14%.	[77]
	Zein, sodium caseinate and quaternary ammonium chitosan	In vitro and vivo	The bioaccessibility of curcumin in free curcumin, uncoated zein–sodium caseinate nanoparticles and positively charged quaternary ammonium chitosan-coated protein nanoparticles is 10.8%, 23.7% and 45.2%, respectively.	[80]
Apigenin	Casein	In vitro and vivo	The bioavailability of apigenin in nanoparticles in rats was 3.0 times higher than that in free group.	[78]
Mulberry anthocyanins	Caseinate and konjac glucomannan	In vitro	After continuous digestion in simulated intestinal juice for 3 h, the cumulative release of nanoparticles was 85.07%, which was higher than 74.30% of free anthocyanins.	[81]
Phloretin	Gliadin and sodium carboxymethyl cellulose	In vitro	The bioaccessibility of phloretin in nanoparticles (55%) was more than twice that of free phloretin (23%) after digestion with simulated gastrointestinal juice.	[82]

**Table 2:** Classification and characteristics of passive targeted nanodelivery systems.

Types	Advantages	Disadvantages
pH-sensitive	This type can solve the premature release of bioactive substances in the gastrointestinal tract and their insufficient accumulation in the target tissue (the small intestine) through the pH difference between the small intestine and stomach.	This type focuses primarily on the impact of chemical barriers corresponding to pH on the delivery system, largely ignoring the effect of other gastrointestinal barriers.
Enzyme-responsive	This type releases bioactive substances in the small intestine after enzyme-related high specific response.	Similarly to pH-sensitive nanodelivery systems, this type also fails to account for the obstruction posed by additional gastrointestinal barriers, particularly physical barriers governing the penetration and absorption mechanisms of bioactive substances.
Mucoadhesive	This type could typically prolong the intestinal residence time by adhering to the intestinal mucus, thereby improving the delivery efficiency.	This type may adhere non-specifically to surfaces to which they are not intended to adhere (e.g., gastric mucosa and intestinal contents). In addition, if they trapped in loose mucus layer may be removed with the renewal of mucus, reducing the absorption of bioactive substances by the human body.
Mucus-penetrating	Compared with mucoadhesive nanodelivery systems, this type may be a more promising strategy to increase the absorption of bioactive substances. Because they are more likely to reach the epithelial cells and subsequently be absorbed.	The gastrointestinal stability of this type needs to be ensured, particularly their structural integrity maintenance during digestion, a critical prerequisite for ensuring mucus-penetrating capability.
Composite	Compared with a single type of delivery system, composite nanodelivery systems that consider multiple barriers have been shown to be more advantageous in the delivery of bioactive substances.	This type still ignores the last barrier to overcome the absorption to a certain extent, namely, the intestinal epithelial cell layer.

**Table 3:** Some single ligand–receptor-mediated targeted nanodelivery systems.

Bioactive Substances	Main Material	Receptors	Model	Main Results	References
Exenatide	DSPE–PEG–FA, LabrafacWL1349, Span80	FA receptor	In vitro and vivo	The bioavailability of the targeted nanocomplex (7.53%) was 1.28 and 2.04 times that of the non-targeted and free exenatide groups, respectively.	[161]
Lutein	Phycocyanin, triphenylphosphonium-modified chitosan, phycocyanin, 3-boronobenzoic acid-modified yeast β-glucan yeast	Dectin-1 receptor	In vitro and vivo	The concentration of lutein in the targeted nanoparticles in the blood and eyeball of rats was 2.54 and 1.82 times higher than that of free lutein, respectively.	[162]
Oridonin	DSPE–PEG2000, LIPOID S100, PLGA, WGA–DOPE	Lectin receptor	In vitro and vivo	The bioavailability of the targeted nanosystem was 9.09 and 1.96 times higher than that of the suspension and non-targeted nanosystem, respectively.	[163]
Ovalbumin	Cationic quaternary ammonium corn starch, carboxymethyl corn starch, GRGDS peptide	Integrin α5β1	In vitro	Compared with the unmodified nanocapsules, the targeted peptide-modified nanocapsules exhibited significantly better ability to target M cells and transport efficiency.	[164]
Insulin	P(GA–co–GAPBAPE), DSPE–PEG–Mal, Fc fragment of Immunoglobulin G	FcRn	In vitro and vivo	The continuous hypoglycaemic ability of targeted nanoparticles was up to 16 h, which was 4 times longer than that of free insulin	[154]
	VB12–chitosan conjugate, calcium chloride, ammonium phosphate	IF receptor	In vitro and vivo	The bioavailability of VB12-modified nanoparticles (26.91%) was 4.3 times that of unmodified nanoparticles.	[165]

Abbreviations: DSPE–PEG–FA, 1,2-distearoyl-sn-glycero-3-phosphoethanolamine-N-(folate (polyethylene glycol) 2000); WGA–DOPE, wheat germ agglutinin-1,2-dioleoyl-*sn-glycero*-3-phosphoethanolamine conjugates; DSPE–PEG2000, 1,2-distearoyl-sn-glycero-3-phosphoethanol-amine-N-methoxy(polyethyleneglycol)-2000; P(GA–co–GAPBAPE), poly(l-glutamic acid-co-l-glutamyl phenylboronic acid pinacol ester); DSPE–PEG–Mal, 1,2-distearoyl-*sn*-glycero-3-phosphoethanolamine-*N*-[maleimide(polyethylene glycol)].

**Table 4:** Some single ligand-transporter-mediated targeted nanodelivery systems.

Bioactive Substances	Main Material	Receptors	Model	Main Results	References
Curcumin	Taurocholic acid–polyethylene glycol 100–monostearate	ASBT	In vitro and vivo	The highest bioavailability of the targeted nanolipids was 15.21 and 65.00 times that of the non-targeted and free curcumin groups, respectively.	[166]
EOFAZ	Bovine serum albumin–dextran sulphate conjugate, sodium deoxycholate	ASBT	In vitro and vivo	The bioavailability of deoxycholic acid-targeted modified nanoemulsion and untargeted modified nanoemulsion was 3.83 and 2.65 times that of the free group, respectively.	[167]
Docetaxel	Dipeptide modified polyoxyethylene stearate, PLGA	PepT1	In vitro and vivo	The bioavailability of targeted nanoparticles was 4.39 and 1.95 times higher than that of docetaxel solution and unmodified solution and unmodified, respectively.	[168]
Insulin, immunoglobin G	Polymer polymerised from three monomers (cationic, anionic, and zwitterionic ones)	PAT1	In vitro and vivo	The bioavailability of free insulin, polymer/insulin and polymer/immunoglobin G nanocomplexes encapsulated in enteric capsules was 1.3%, 16.9% and 12.5%, respectively.	[169]
Doxorubicin	Linolenic acid conjugated chitosan	Fatty acid transporter 4	In vitro and vivo	The bioavailability of targeted nanomicelles in rats was 1.66 times higher than that of free doxorubicin.	[170]
Insulin	Biotin grafted chitosan, hyaluronic acid	Sodium-dependent multivitamins transporter	In vitro and vivo	Free insulin did not reduce blood glucose, and the highest hypoglycaemic ability of targeted nanocomposites was 1.92 times that of non-targeted nanocomposites.	[171]
	DSPE–PEG–Fru, PLGA, DSPE–PEG	Glucose transporter 2	In vitro and vivo	The bioavailability of fructose- targeted nanoparticles was 2.35 and 3.78 times that of non-targeted and free groups, respectively.	[172]

Abbreviations: EOFAZ, essential oil from *Alpinia zerumbet* Fructus; DSPE–PEG–Fru, fructose-modified 1,2-distearoyl-sn-glycero-3-phosphatidylethanolamine-PEG.

## Data Availability

No new data were created or analysed in this study. Data sharing is not applicable to this article.
